# Effects of captivity, diet, and relocation on the gut bacterial communities of white‐footed mice

**DOI:** 10.1002/ece3.6221

**Published:** 2020-04-03

**Authors:** Pauline van Leeuwen, Nadia Mykytczuk, Gabriela F. Mastromonaco, Albrecht I. Schulte‐Hostedde

**Affiliations:** ^1^ Department of Biology Laurentian University Sudbury ON Canada; ^2^ Conservation Genetics Laboratory University of Liège Liège Belgium; ^3^ Vale Living with Lakes Centre Laurentian University Sudbury ON Canada; ^4^ Reproductive Physiology Toronto Zoo Scarborough ON Canada

**Keywords:** 16S rRNA gene, captivity, conservation biology, diet, gut microbiota, reintroduction, wild mice

## Abstract

Microbes can have important impacts on their host's survival. Captive breeding programs for endangered species include periods of captivity that can ultimately have an impact on reintroduction success. No study to date has investigated the impacts of captive diet on the gut microbiota during the relocation process of generalist species. This study simulated a captive breeding program with white‐footed mice (*Peromyscus leucopus*) to describe the variability in gut microbial community structure and composition during captivity and relocation in their natural habitat, and compared it to wild individuals. Mice born in captivity were fed two different diets, a control with dry standardized pellets and a treatment with nonprocessed components that reflect a version of their wild diet that could be provided in captivity. The mice from the two groups were then relocated to their natural habitat. Relocated mice that had the treatment diet had more phylotypes in common with the wild‐host microbiota than mice under the control diet or mice kept in captivity. These results have broad implications for our understanding of microbial community dynamics and the effects of captivity on reintroduced animals, including the potential impact on the survival of endangered species. This study demonstrates that ex situ conservation actions should consider a more holistic perspective of an animal's biology including its microbes.

## Graphical abstract

This study simulated a captive breeding program with white‐footed mice to describe the variability in gut microbial community structure and composition during captivity and relocation in their natural habitat and compared it to wild individuals. Captive‐reared mice were separated into two groups: one with standard pellets and the other with nonprocessed food that is a captive version of a wild diet; they were then relocated in the wild. Overall, the immediate external environment has the strongest effect on the gut bacteria, followed by the diet in captivity and relocated mice that had the nonprocessed diet had more bacterial phylotypes in common with the wild‐host microbiota than any other group, meaning that captive diet has a strong impact on the gut bacteria of animals even after relocation and it should be taken into account for conservation practices.

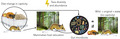

## 1 INTRODUCTION

The reintroduction and relocation of individuals in the context of species conservation faces many challenges (Fischer & Lindenmayer, 2000; Game, Meijaard, Sheil, & Mcdonald‐Madden, 2014; Seddon, Armstrong, & Maloney, 2007), including the fact that individuals released from captive breeding programs often struggle to thrive in their natural habitats (Gilbert, Gardner, Kraaijeveld, & Riordan, 2017; Willoughby & Christie, 2019). These difficulties may be caused by adaptations acquired from generations of captivity (Schulte‐Hostedde & Mastromonaco, 2015; Snyder et al., 1996), and disease (Kołodziej‐Sobocińska, Demiaszkiewicz, Pyziel, & Kowalczyk, 2018; Viggers, Lindenmayer, & Spratt, 1993) and/or the inability to transition to a native diet (Jules, Leaver, & Lea, 2008; Kleiman, 1989).

It has been increasingly recognized that host‐associated microbes should be considered in wildlife management practices, particularly in the context of conservation (Amato, 2013; Bahrndorff, Alemu, Alemneh, & Nielsen, 2016; Redford, Segre, Salafsky, Rio, & Mcaloose, 2012; Stumpf et al., 2016; Trevelline, Fontaine, Hartup, & Kohl, 2019; West et al., 2019). Animals provide numerous ecological niches for microorganisms, such as bacteria, archaea, protozoa, fungi, and viruses. These communities of microbes in a host are collectively known as the microbiota. The gut microbiota can play a role in host development, digestion, immunity, and behavior (McKenney, Koelle, Dunn, & Yoder, 2018; Suzuki, 2017) and can therefore influence the survival of relocated animals. Host‐associated microbiota are highly dynamic communities, and disrupting their equilibrium can lead to negative direct or indirect effects on their host (Hooks & Malley, 2017; reviewed in West et al., 2019) such as impaired immune function and metabolic disorders (Clayton et al., 2016; Krynak, Burke, Martin, & Dennis, 2017; Rosshart et al., 2017; Wasimuddin et al., 2017).

The external environment of a host (Schmidt, Mykytczuk, & Schulte‐hostedde, 2019), its diet, and genetics (Campbell et al., 2012; Spor, Koren, & Ley, 2011) are all known to modify the gut microbiota. Housing facilities such as zoos where captive breeding programs are held provide intense veterinary care, sanitized enclosures, a standardized diet, and reduced sexual selection. Captivity has been shown to alter the microbiota of animals compared to wild counterparts (Borbón‐García, Reyes, Vives‐Flórez, & Caballero, 2017; Clayton et al., 2016; McKenzie et al., 2017; Wasimuddin et al., 2017). The majority of the studies show similar trends: a decrease in bacterial phylotype richness (or α‐diversity) among captive individuals compared to their wild conspecifics, as well as differences in community composition (or β‐diversity) between the groups. However, some host species show an opposite pattern (Frankel, Mallott, Hopper, Ross, & Amato, 2019; Greene et al., 2019; McKenzie et al., 2017), postulating that the gut microbiota of group taxa respond differently to captivity, mainly through their feeding strategy and gut physiology. Differences observed in gut microbial communities have largely been attributed to altered diets in captivity that can also lead to the extinction of microbial niches and functions in the host's gut over multiple generations in captivity (Sonnenburg et al., 2016). Standardized diets are generally composed of simple fibers in low quantity compared to carbohydrates. A loss of microbial taxa taking part in the digestion of fibers in captivity has been linked to disease as a short‐term disadvantage to hosts (Amato et al., 2016; Krynak et al., 2017; Rosshart et al., 2017) and could also, in the long run, be a disadvantage when hosts are relocated to their natural habitat. In general, there is extensive gut microbiota variation when an animal fed on a more animal‐based or plant‐based diet for humans and mice, compared to a balanced diet from various food sources (Heiman & Greenway, 2016). The consumption of a diverse diet avoids the loss of crucial microbial function linked to a specific food item in the case of omnivorous host. This has been demonstrated through the evolution of human lifestyles. Human microbial communities have been shaped through changes from hunter‐gatherer and nomad societies to farming, sedentary, and urban lifestyles. Especially in Western diets, the lack of fibrous food items and the increased consumption of processed foods have resulted in a reduction of gut bacteria diversity that has been implicated in many diseases linked to impaired immune responses and metabolic disorders (Kolodziejczyk, Zheng, & Elinav, 2019). It is therefore essential to study the effects of changes in diet during captivity on the gut microbiota among different host taxa with variable ecological niches, being dietary generalists or specialists, omnivorous or herbivorous, for example. Previous work has suggested that a change to a more fibrous and less processed diet in captivity changes the gut microbiota compared to a standard diet, but it does not make the gut microbiota of captive animals more similar to their wild counterparts (Allan et al., 2018; Cabana et al., 2019). However, the impacts of diet change on gut bacteria remain to be investigated during animal relocation.

Few studies have shown how host‐associated microbiota vary between captivity and relocation into a natural habitat and mainly focused on the impacts of place of birth and the immediate environmental exposure (Chong et al., 2019; Metcalf et al., 2017; Schmidt et al., 2019; Yao et al., 2019). Even less studies looked at the effect of captive diet on the gut microbiome during animal relocation (Martinez‐Mota, Kohl, Orr, & Dearing, 2019). Overall, animals born in captivity have lower α‐diversity and more differences in microbial communities compared to animals born in nature reserves or in the wild (Metcalf et al., 2017). Furthermore, deer mice (*Peromyscus maniculatus*) born in captivity and later released had gut microbial communities closer to their wild counterparts compared to animals that stayed in captivity (Schmidt et al., 2019). Since the literature gap of the effects on gut bacteria of captive diet during relocation remains unaddressed, we focused on the impact of captive diet for the gut bacteria during the relocation process in a generalist species with an omnivorous diet. We hypothesized that diet during captivity will affect the gut microbiota of the host during the relocation process and can maximize the reintroduction success of an animal back into its natural habitat. We predict that a wild‐like and nonprocessed diet in captivity would foster the recovery of a wild‐like microbiota after the animal is relocated in their natural habitat, compared to a standard captive diet composed of pellets. Therefore, captive diets reflecting a wild diet could have benefits associated with improved degradation of food items by microbes, echoing higher microbial diversity in the gut of mice under a wild‐like diet compared to a standard and processed diet in captivity. This study was conducted on the gut bacterial communities of the white‐footed mouse (*Peromyscus leucopus*), an omnivorous rodent native to Ontario (Canada) that feeds primarily on insects, seeds, nuts, and fruits, just like its closely related species, *P. maniculatus* (Wolff, Dueser, & Berry, 1985). *Peromyscus leucopus* does not face major threats of extinction, but its large distribution, short generation time, and high capture–recapture rate in general make it an adequate model to study gut microbiota variation across a short period of time to simulate a captive breeding program for reintroduction purposes.

## 2 MATERIALS AND METHODS

### 2.1 Sample collection

All methods were approved by the Institutional Animal Care and Use Committee (IACUC) at Laurentian University and by the Toronto Zoo Animal Care and Research Committee (ACRC) under the reference 2018‐05‐02. White‐footed mice (*P. leucopus*) were trapped on the grounds of the Toronto Zoo (ON, Canada) using Longworth traps. Trapping occurred five nights a week during the breeding season of June to mid‐September 2018. Each mouse was identified with unique numerical tags (National Brand and Tag Co.) and weighed. Wild juveniles were detected by the color of their fur (gray) and their weight (<15 g) and were excluded from the study. Fecal samples were collected directly from the animal using flamed and 70% ethanol‐sterilized tweezers and stored in sterile microcentrifuge tubes in a −20°C freezer until DNA extraction.

### 2.2 Experimental design

**FIGURE 1:**
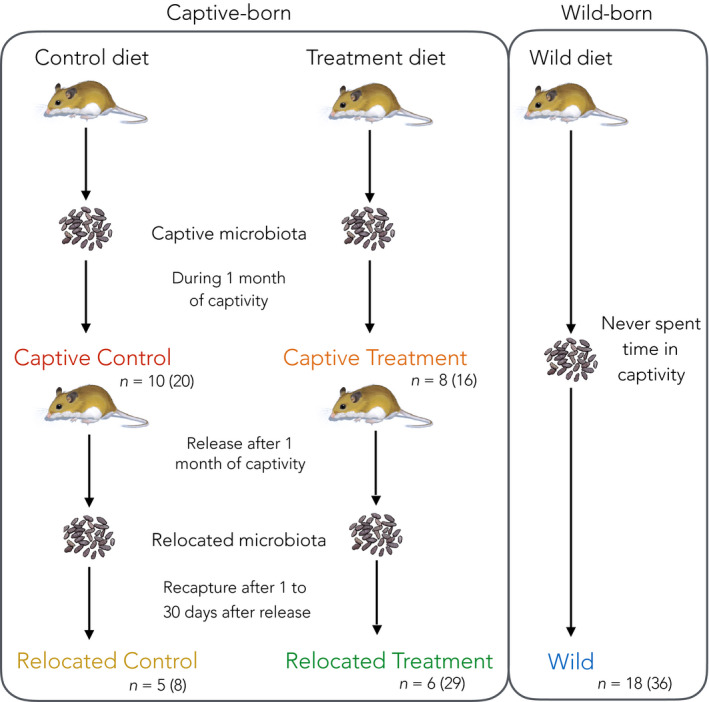
Experimental design for study conditions and fecal collection. Each color represents the different mice groups, with *n* the number of individuals and in brackets the number of fecal samples overall

Pregnant dams were brought into an animal holding unit within the Wildlife Health Centre of the Toronto Zoo (ON, Canada) in June and July. Twenty‐one days after parturition, the offspring were separated from their dam and housed in individual cages ( $\bar{x}=4$ , 5 juveniles/L). Animals were placed in individual disposable plastic cages (37.3 × 23.4 × 14.0 cm; Innovive) with cut straw from wheat, nesting material (Ancare Nestlets), and PVC tubes for environmental enrichment. Food and water were provided ad libitum. The mothers were fed with standard rodent chow. To limit bias from any maternal effects, one half of each litter was given a control diet, becoming part of the Captive Control group (CC) and the other half received the treatment diet, belonging to the Captive Treatment group (CT). For all the groups in this experimental design, the first letter corresponds to the external environment of the mouse at the time of fecal collection and the second letter to the diet they received during their time in captivity (Figure 1). The control group received the same diet as their mothers, and the treatment group received a diet composed of sunflower seeds, diced apples, crushed walnuts, mealworms, and crushed corn in equal proportions. Each animal received its respective diet and was kept in these conditions for 30 days until they reached sexual maturity. Fecal samples were collected 8 days and 1 day prior to release for each individual (Table 1). Those samples represent the Captive Control (CC) and Captive Treatment (CT) study groups. All offspring were then released at one of three locations on the grounds of the Toronto Zoo. Fecal samples were collected from all wild adults trapped in this period at least twice, 7 days apart (W for Wild experimental group; Table 1). Dams that gave birth in captivity were released but were excluded from this study group. For each recapture of released offspring, becoming, respectively, the Relocated Control group (RC) and the Relocated Treatment group (RT) depending on their diet in captivity, fecal samples were collected opportunistically, and all were included in the sampling design. It occurred that some relocated mice were never recaptured, and others were recaptured multiple times. Some wild and relocated mice experienced botfly *Cuterebra* sp. infection during the sampling period. It is characterized by subcutaneous swelling around the genital area (warble) of the mice and the movement of the infectious larvae in this swelling. The presence or absence of infection was considered in the sampling and analysis of the data.

**TABLE 1:** Individual mice and related samples involved in the study

(A)						
Mouse ID	Sex	Dam ID	Diet	*Cuterebra* sp. (after relocation)	Samples collected in captivity	Samples collected after relocation
691	M	D	Control	Presence	2	4
999	F	A	Control	Absence	2	1
1353	F	B	Control	Absence	2	1
1995	M	B	Control	N/A	2	0
6110	F	A	Control	N/A	2	0
7221	F	C	Control	Absence	2	1
7471	M	C	Control	Absence	2	1
8022	M	C	Control	N/A	2	0
8624	M	D	Control	N/A	2	0
54100	F	A	Control	N/A	2	0
Total by group					Captive control: 20	Relocated control: 8
496	M	D	Treatment	Presence	2	8
1407	F	A	Treatment	N/A	2	0
6057	F	A	Treatment	Absence	2	1
7631	F	C	Treatment	Absence	2	4
7975	M	C	Treatment	Absence	2	9
8362	F	D	Treatment	Absence	2	1
8887	M	C	Treatment	Presence	2	6
9798	M	D	Treatment	N/A	2	0
Total by group					Captive treatment: 16	Relocated treatment: 29

(B)			
Mouse ID	Sex	*Cuterebra* sp.	Samples collected
52	M	Presence	2
58	M	Presence	2
59	F	Presence	2
65	M	Presence	2
89	F	Presence	2
92	M	Presence	2
93	F	Presence	2
124	F	Presence	2
146	M	Absence	2
1	M	Absence	2
3	M	Absence	2
18	M	Absence	2
51	F	Absence	2
73	F	Absence	2
82	F	Absence	2
Total			Wild: 36

Note

(A) Mice and related samples that were born in captivity and were under the two different diets. Once relocated in the wild, some mice were not recaptured and others have been recaptured more than once. All samples collected from the recaptured mice have been included in the study. (B) Mice and related samples collected from the wild that never experienced captivity. Some had botfly infection, and its effect has been taken into account in the later analysis.

### 2.3 DNA extraction and sequencing

Gene amplicon sequencing was used to study the bacterial communities. DNA extractions from the fecal samples collected were conducted using the Stool DNA Isolation Kit (Norgen Biotek Corp) following the manufacturer's instructions. Two blank extractions were made to control for contamination during the extraction process. After DNA extraction, the targeted gene for taxonomic affiliation (16S rRNA gene) was amplified through polymerase chain reactions (PCRs). The library preparation and sequencing were performed by Metagenombio Inc., as well as the demultiplexing of the sequence reads. Using their designated library protocol, 2 × 300 bp paired‐end sequencing was completed using broad bacterial primers of the region V4 of the 16S rRNA gene (515F‐806R) using an Illumina MiSeq platform (Illumina Biotechnology Co.).

### 2.4 Bioinformatics

The quality controls of the already demultiplexed paired‐end sequence reads were performed through the software FastQC (Andrews, 2010). Sequence reads denoising and amplicon sequence variants (ASVs) picking steps were done with the QIIME2 tool (Bolyen et al., 2018; v. 2019.1), using the DADA2 pipeline (Callahan, Mcmurdie, & Holmes, 2017; Callahan et al., 2016). ASVs—or also referred to as bacterial phylotypes—were then screened to the 97% 16S rRNA gene full‐length reference sequences from the Silva v.132 database (Pruesse et al., 2007) for taxonomical association using the VSEARCH classifier implemented in QIIME2 (Bokulich et al., 2018). Sequence alignment and phylogeny building were conducted in QIIME2 for the construction of a generalized UniFrac distance matrix (*α* = .5; Chen et al., 2012). The cumulative sum scaling (CSS) method was used to normalize the data using the *metagenomeSeq* package (Paulson, Colin Stine, Bravo, & Pop, 2013) in R (R version 3.5.2, R Core Team, 2018). It can decrease the fold difference in sampling depth and avoid the rarefying of counts (McMurdie & Holmes, 2014; Paulson et al., 2013; Weiss et al., 2017).

### 2.5 Statistical analysis for α‐diversity of gut bacteria between study groups

All statistical analyses were conducted in R (R version 3.5.2, R Core Team, 2018) using the *phyloseq* (McMurdie & Holmes, 2013) and *microbiome* packages (Lahti, 2017) for manipulation of data. Fisher's diversity index and Simpson evenness index of the phylotypes in each sample were used as metrics to measure the α‐diversity of gut bacteria between samples. Differences in the indexes according to study group, sex, date, infection status, place of birth, and interactions were analyzed using linear mixed models with a restricted maximum likelihood estimation approach with mouse ID and dam ID as random factors, using the lmer function in the *lme4* package in R. The study group variable was first considered as two distinct factors: diet and environment. If the two factors and the interaction between the two had a significant effect on the variable, they were combined as “study group. ANOVAs with Satterthwaite's method were run on these models, as well as post hoc Tukey method for *p*‐value adjustments was conducted to investigate differences between groups. Normality of residuals was validated using the Shapiro–Wilk test. The significance cutoff was set to *p*‐value < .05 for each test.

### 2.6 Statistical analysis for β‐diversity of gut bacteria between study groups and differential abundance

A generalized UniFrac distance matrix between samples (Chen et al., 2012) was used to investigate differences in gut microbial communities between groups, sex, for maternal effect, and *Cuterebra* infection. This metric takes into account the differences in phylogenetic distance and abundance of each bacterial community between samples, pairwise. A PERMANOVA model adonis from the *vegan* package was constructed with 9,999 permutations with reported F, R2, and *p*‐values, to determine whether there were significant differences between the study groups (Oksanen et al., 2019). Mouse identification tag was used as stratification to account for repeated measures and the model included the sex of the individual, the dam ID, infection status, interactions between those factors, and the study groups in a similar way as the α‐diversity analysis. A detrended correspondence analysis (DCA) was conducted to detect the gradual structure in the samples. As multiple samples come from the same individual across time and different environments, the transition from captivity to the wild can be considered as a gradient. The fact that the correspondence analysis is detrended improves the dispersion of point in the ordination of the samples by generalized UniFrac distances and removes the arch effect. Finally, a minimum spanning tree was constructed using the *phyloseqGraphTest* package (Callahan et al., 2016).

The differential abundance analysis was conducted on the ASVs that were present in more than 5% of all the samples and that had a relative abundance of more than 5% among all taxa. It corresponds to the core microbiota of the dataset, represented by 653 phylotypes. The phylotype abundance analysis was made using the *DESeq2* package (Love, Huber, & Anders, 2014), using a negative binomial Wald test to test significance in contrast between each study group. Only phylotypes with a significance level (*α*) below .001 after false discovery rate (FDR) corrections were considered using the Benjamin–Hochberg method .

**TABLE 2:** Summary of significantly enriched phylotypes among the study groups from the DESEq2 analysis

Phylum	Family	Genus	Captive Control		Captive Treatment		Captive Control and Captive Treatment		Relocated Control		Captive Control and Relocated Control		Relocated Control and Relocated Treatment		Relocated Treatment		Relocated Treatment and Wild		Wild		Total
			Reduced	Enriched	Reduced	Enriched	Reduced	Enriched	Reduced	Enriched	Reduced	Enriched	Reduced	Enriched	Reduced	Enriched	Reduced	Enriched	Reduced	Enriched	
Bacteroidetes	Muribaculaceae	Uncultured bacterium	1		3		1		5				1		1					2	14
	Prevotellaceae	Nonassigned																		1	1
		Prevotellaceae UCG‐004	1																		1
	Rikenellaceae	Alistipes	2								1										3
	Rs‐E47 termite group	Unknown													1						1
Epsilonbacteraeota	Helicobacteraceae	Helicobacter							1												1
Firmicutes	Lachnospiraceae	[*Eubacterium] xylanophilum* group					1		1											1	3
		Coprococcus 2	1				1														2
		Lachnospiraceae NK4A136 group	1		2		3		4		3							4	2	2	21
		Lachnospiraceae UCG‐001			1				1												2
		Roseburia							1												1
		Unknown	2				2		1					1	1					1	8
	Ruminococcaceae	Uncultured	1																		1
	Staphylococcaceae	Staphylococcus																	2		2
Tenericutes	Mycoplasmataceae	Ureaplasma																		1	1
Total			9	0	6	0	8	0	14	0	4	0	1	1	3	0	0	4	4	8	62

Note

For each mice group, a number of unique phylotypes are reduced or enriched in abundance compared to all the other groups and belong to the different taxa on the left. Some phylotypes were also common in two groups compared to the others.

## 3 RESULTS

A total of 874,824 sequences of 3,206 bacterial phylotypes (or ASVs) were identified from the 109 samples after the removal of the features present in the two blank samples to avoid DNA extraction bias (mean sequences by samples: 7,043; min: 4,280; max: 14,927). In total, 36 mice were included in the study (*n* = 18 W; *n* = 10 CC; *n* = 8 CT; *n* = 5 RC; *n* = 6 RT) and 109 fecal samples were obtained from those mice (*n* = 36 W; *n* = 20 CC; *n* = 16 CT; *n* = 8 RC; *n* = 29 RT). The low number of mice in the RC and RT groups is due to the fact that the other mice released were not recaptured after relocation (Table 1).

### 3.1 α‐diversity of gut bacteria between study groups according to the host's birthplace

**FIGURE 2:**
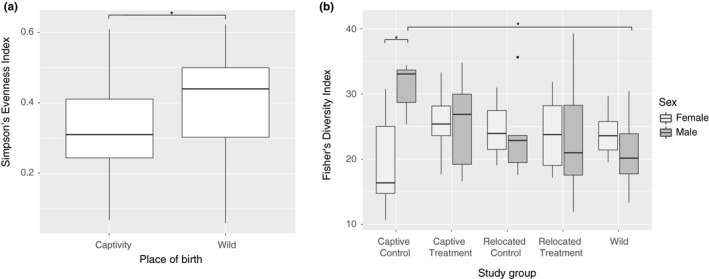
Boxplots representing changes in (a) Simpson's evenness index variation of the gut microbiota depending on host's birthplace, and of (b) Fisher's diversity index of gut microbiota among the different study groups and according to the sex of the host. * represents the *p*‐value meeting the standard cutoff of *p* < .05

Some relocated and wild animals were sampled during a *Cuterebra* infection (*n* = 26 infected; *n* = 79 noninfected; Table 1), but they were not treated separately in the statistical analysis as it explained 1% of the community variation (ANOVA: *F* = 0.0171; *p* = .896237). There was a significant difference in terms of phylotype evenness between mice born in captivity and in the wild (Simpson's evenness index: *F* = 2.785; *p* = .01877; Figure 2a), so that mice born in captivity carry gut communities less uniform in phylotype abundance. The interaction of host sex and study group also had a significant impact on the gut bacterial phylotype richness (Fisher's index: *F* = 6.2087; *p* = .006176; Figure 2b). Male mice from the CC group had significantly higher gut bacteria phylotype richness compared to females from the same group (Tukey: *F* = 4.4974; *p* = .031458) or compared to males from the wild (Tukey: *F* = 4.4974; *p* = .03992457; Figure 2b).

From the fecal samples collected, the gut microbial communities of wild mice contained 834 unique phylotypes (5.3% of their gut bacteriome), which is more than captive mice (586, 3%) and relocated animals (525, 1.5%). Relocated and wild mice had 250 common bacterial phylotypes in their gut, which represent a higher proportion (8.8%) than the 238 phylotypes common between relocated and captive (7.3%), and between wild and captive individuals (141, 2.7%). Overall, the three groups had 573 phylotypes in common (71.3%). Similar proportions were found between gut bacteria of wild mice and mice that had the control and treatment diets.

### 3.2 β‐diversity of gut bacteria between study groups

**FIGURE 3:**
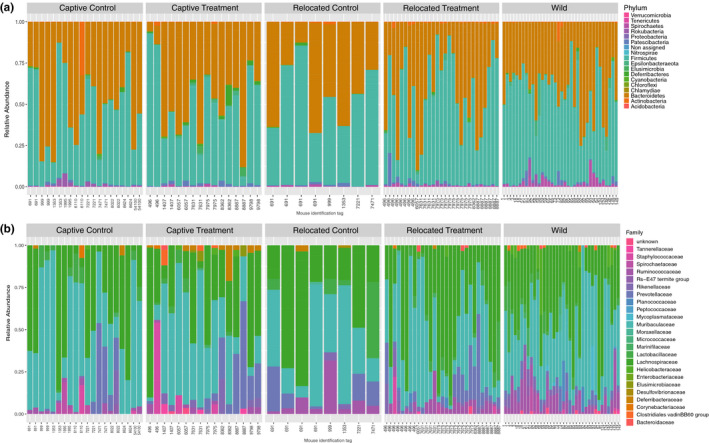
Compared relative abundance of bacterial taxa for each study group of mice in the study (taxa showing less than 0.1% of relative abundance were not included). In each group, samples are sorted by mouse individual and by date. Stacked barplots showing the relative abundance at the (a) phylum and (b) family levels for gut bacteria among the study conditions

As expected in mammal gastrointestinal tracts, all samples were dominated by the *Firmicutes* and *Bacteroidetes* phyla (Figure 3a; McKenzie et al., 2017). Males and females were not treated separately in subsequent statistical analyses. The gut bacterial community composition of male and female mice considered in the study (adonis: *F* = 3.6162; *R* ^2^ = .02795; *p* = .23676) was not significantly different and all explained around 3% of the variation. *Cuterebra* infection did not have a significant effect on gut community composition as it explained 1% of the community variation (adonis: *F* = 1.4469; *R* ^2^ = .01060; *p* = .269730), neither was litter affiliation (adonis: *F* = 3.9089; *R* ^2^ = .09062; *p* = .12887).

**FIGURE 4:**
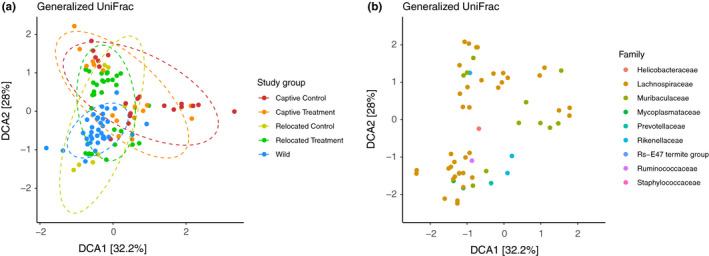
The detrended component analysis (DCA) ordination method was used on generalized UniFrac distances between samples to visualize differences of microbiota in the experimental groups. (a) Microbiota of mice from the different experimental groups with 90% confidence limit ellipses. (b) Plot showing the 62 significant bacterial phylotypes that were differentially abundant in the experimental groups after DESeq2‐based analysis, according to the DCA ordination method

**FIGURE 5:**
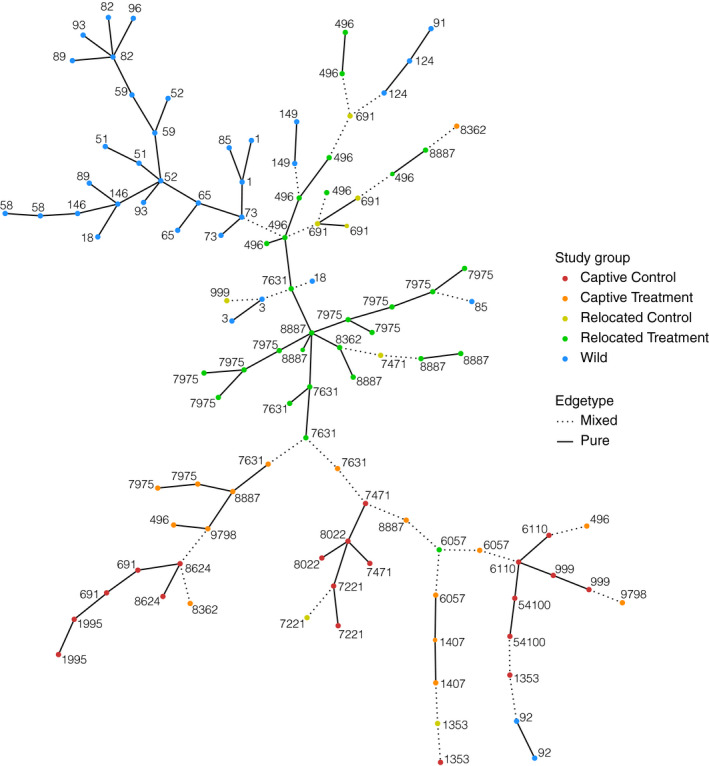
Minimum spanning tree of samples based on generalized UniFrac distances on all phylotypes. From 9,999 permutations, this tree was obtained with 73 pure edges on 104 with permutation *p*‐value < .0001. Colors represent the study groups, and each sample is named after the mouse identification tag

Fecal microbiota differed more in composition between captive and wild mice than between relocated and wild mice (Figure 3; Figure 5; Figure S1; adonis: *F* = 2.9232; *R* ^2^ = .08742; *p* = .000999). Detrended component analysis (DCA) on generalized UniFrac distances demonstrated differences of microbiota between the study groups (Figure 4a). The ordination plot shows that microbiota from captive and wild mice are more distant to each other, compared to relocated (RC and RC) and W mice. A minimum spanning tree generated using a generalized UniFrac distance matrix also shows that microbiota from wild mice are closer to microbiota of relocated animals compared to the ones from captive mice (Figure 5). Study groups tend to aggregate together, and it was mainly driven by different abundances of taxa in the families *Lachnospiraceae*, *Muribaculaceae*, for example, as shown by a split biplot (Figure 4; Figure 5). When running the analysis on samples coming from CC and CT mice only, differences among the samples were explained by the diet but also through litter affiliation (Figure 5; adonis: *F* = 1.4141; *R* ^2^ = .01170; *p* = .037962).

When considering the diets in the relocated and captive groups, the microbiota from RT mice was overall more closely related to the W mice microbiota than the RC mice microbiota (adonis: *F* = 2.9232; *R* ^2^ = .08742; *p* = .000999). The microbiota from mice of the CT group was then more closely related to microbiota from mice of relocated groups (RC and RT) and W compared to the CC group (Figure 5).

### 3.3 Differential abundance among groups

**FIGURE 6:**
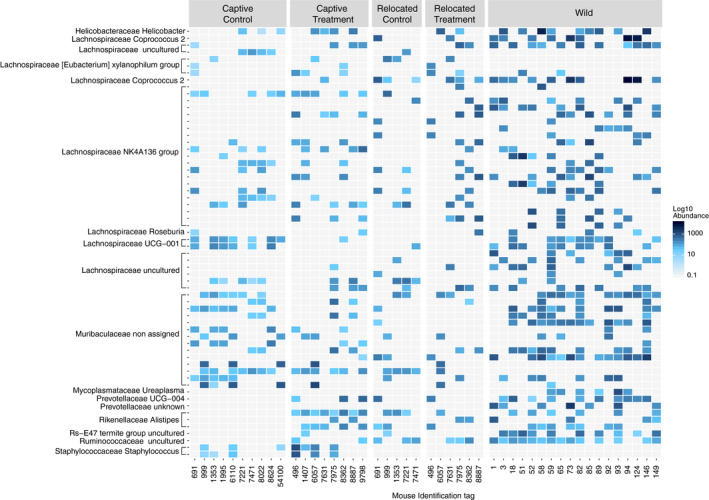
Heatmap representing the results of the differential abundance analysis. Samples on the *x*‐axis are grouped by mouse identification tag and by study group. The different colors represent the abundance on a log10 scale of each significantly enriched phylotype (median from all samples by mouse). Each phylotype on the *y*‐axis is named by family and genus

The assessment of the differential abundance of bacterial phylotypes using a negative binomial Wald test was conducted on the core microbiota of 653 phylotypes. From those, 62 from four phyla varied significantly among the study groups (*Bacteroidetes*, *Espilonbacteraeota*, *Firmicutes*, and *Tenericutes*; Figure 6; Table 2). 60% and 22% of the phylotypes with differential enrichment across groups, respectively, belonged to the *Lachnospiraceae* and *Muribaculaceae* families. Mice from the CC and RC groups had the greatest loss in abundance in gut phylotypes compared to the other study groups (Table 2; respectively, nine for CC and 14 for RC). Overall, the RT group was the only group that had significant phylotypes enriched and in common with gut communities from W mice compared to the other study groups (Figure 6; Table 2).

## 4 DISCUSSION

### 4.1 α‐diversity of gut bacteria between study groups

We compared the gut microbiota of mice in captivity under different diets, after relocation, and in the wild. The structures of the gut microbial communities in terms of phylotype richness were similar among study groups, with significantly higher phylotype richness only observed in the gut microbial communities of male CC mice compared to females and to wild males. This result is not common on gut microbiome studies in wildlife and explains no or little variation (Schmidt et al., 2019; Wasimuddin et al., 2017). In that case, captivity could have a sex‐specific effect on the gut microbiota of *P. leucopus*. However, we found no apparent differences in community composition on beta diversity analyses; these results could thus be an artifact from low sample sizes.

The structure of the microbiota in terms of evenness is more uniform in wild‐born mice than captive‐born mice that have more disparate microbial communities. Similar results were found in studies including the place of birth as a factor of variation in gut microbiota for horses and deer mice (Metcalf et al., 2017; Schmidt et al., 2019). Kohl and Dearing (2014) also observed that evenness decreased with time spent in captivity in desert woodrats. It has been hypothesized that this difference could be due to lasting founder effects of colonization of the gut by microbes during the early life of the host. The natural habitat would be the source of more diverse bacterial phylotypes (interactions with more species, diverse substrates and diets, seasonality, and no antibiotic treatments) compared to captivity. However, the opposite trend was observed in Andean bears and red pandas (Borbón‐García et al., 2017; Kong et al., 2014). Host diet, phylogenetics, and position in trophic networks could thus be important factors to consider. Overall, the evenness in bacterial communities can affect the subsequent response to disturbances and is known as the insurance hypothesis (Wittebolle et al., 2009), suggesting that place of birth may have on impact on host survival from the gut microbiome aspect. However, it is worth mentioning that differences in diversity indexes between wild and captive mice might be due to the fact that there is no knowledge about relatedness between animals of the W group, whereas captive‐born animals come from a handful of litters that can have an impact on the gut microbiota (Spor et al., 2011).

### 4.2 β‐diversity of gut bacteria between study groups and differential abundance among study groups

From the β‐diversity analysis, we observed that RT and RC individuals were the closest to their wild counterparts in terms of microbial structure and composition than CC and CT animals (Figures 4 and 5). This would imply that the immediate environment has a strong effect on gut microbiota composition. Once the individuals are relocated in their natural habitat, the environment becomes the main source for microbes’ horizontal acquisition, in both external exposure but in diet as well (Colston, 2017). Therefore, the captive diet seems to have a smaller impact compared to external exposure but appears to have lasting effects on the gut microbiota, since it influences its composition and structure even 1 month after relocation (Figure 6).

The reduced influence of the diet compared to the external environment is also reflected in specific phylotype abundances. Similar to Schmidt et al. (2019), the *Lachnospiraceae* family is differently distributed between the gut microbiota of captive, wild, and relocated animals. Although they are present in all groups, *Lachnospiraceae* phylotypes are mostly enriched in the W and RT groups rather than the CC, CT, and RC groups. Maurice et al. (2015) examined the variation of *Lachnospiraceae* in wild *Peromyscus* species. They hypothesized that seasonal variation in the abundance of this taxon is linked to a diet shift from insects to seeds in mid‐summer because these bacterial groups support the degradation of complex plant materials. These taxa seem to play a role in the degradation of butyrate during fiber degradation that promotes colonocyte health, immune defense, and anti‐inflammatory action, reducing the risk of developing metabolic disorders that are a growing concern in captive populations (Meehan & Beiko, 2014; Vijay‐Kumar et al., 2010). However, in our study, the abundance of the *Lachnospiraceae* family is stable between the study groups but, at a lower taxonomical level, genera abundances within this family seem to differ. The *Lachnospiraceae NK4A136 group* had the highest variation between groups: increasing in RT and W mice and decreasing in the other groups. Not much is known of this genus, but it is associated with the digestive tract of mammals, using carbohydrates and producing short‐chain fatty acids (Meehan & Beiko, 2014). Further studies targeting the *Lachnospiraceae* groups would be needed to investigate to which extent there is variation in these taxa between the study groups and their role in the mouse gut.

However, the *Lachnospiraceae* taxa seemed to be absent or reduced in abundance in the gut of the RC mice and those hosts may have loss the beneficial microbial function linked to these taxa. The fact that the RC group had the highest number of phylotype abundance reduction is another indicator that standardized pellets might not be adequate for animal relocation from the gut bacteria perspective. Few studies to date already advocate for a transitional period between captivity and relocation to foster reintroduction success (Yao et al., 2019), and our results recommend similar practices for generalist species like *P. leucopus*. We encourage the production of similar work on hosts with different ecological niches and gut physiology among different taxa, such as Martinez‐Mota et al. (2019) that demonstrated similar results than this study on specialist woodrats.

The *Muribaculaceae* family from the *Bacteroidetes* phylum also followed a similar pattern in terms of variation in abundance: It decreased for some phylotypes in all groups, mostly in the gut of RC mice, and only increased in some phylotypes in the gut of W individuals. A targeted analysis on this taxon would be necessary to understand which exact phylotype varies in abundance. This family, previously named S24‐7, is a dominant bacterial group from the mouse gut. It takes part in the degradation of carbohydrates and produces enzymes involved in the degradation of plant glycans like pectin (Ormerod et al., 2016). Pectin is highly present in apples so it could explain the presence of this bacterial group in treatment individuals, but there is no particular enrichment of this taxon in the gut of the CT and RT groups. This could be explained by the presence of other fibrous food items in the wild mice diet and therefore encourage the optimization of the treatment diet.

Overall, this study reports complementary results advocating that captivity does have an impact on the gut microbial communities of generalist rodents like *P. leucopus* after relocation in their natural habitat. Moreover, altered diets in captivity contribute to those effects. Analogous to Sonnenburg et al., (2016), mice subjected to standard low‐fiber diet recovered less microbiota diversity than mice fed with a high‐fiber, less processed diet. However, the diversity was not in terms of total phylotype richness but in terms of common bacterial groups with the wild “original” state of the microbiota. The generalization that captivity induces an imbalanced microbiota linked to negative effects on the host should be considered with caution, because it can depend on the taxonomy and ecology of the host, as demonstrated by Greene et al. (2019) and Frankel et al. (2019).

It is also worth mentioning that across the studies comparing the gut microbiota of captive and wild animals, some enclosures allow access to open areas, social interaction, and enrichments that favor exposure to the natural habitat of the species (Clayton et al., 2018; Greene et al., 2019). This could shift the gut microbiota of these animals toward a wild‐like state; However, this was not the case in our study and microbiota variation between CC, CT, RC, and RT mice could only be due to diet. Our study demonstrates that for the relocation of generalist rodents, it is not only a matter of captivity itself and external exposure, but also about diet manipulation. Even if the treatment diet might not reflect all the aspects and components of a wild diet for *P. leucopus* and could lead to nutrient deficiency over a long period of time, it is more adequate than standardized pellets for supporting microbiota composition of mice after relocation. Further work on gene expression in the microbiota and on the host's survival should be undertaken to understand the long‐term effects of diet and microbiota variation once an animal is relocated.

It is worth mentioning that the recapture rates between the two relocated mice groups were different. The 75% of RT mice released were recaptured 29 times compared to only eight times for the 50% of released RC group (Table 1). This could be a survival rate indicator, but other ecological factors such as dispersal and predation need to be considered. White‐footed mice are the prey of many animals such as the eastern screech‐owl (*Megascops asio*) that was seen on site, and the persistent presence of raccoons (*Procyon lotor*) that disrupted traps and predated on mice (personal observation) might also account for the low recapture rate of RC mice. One explanation could be that RC mice have been more predated than RT mice because of microbiota‐induced behavior (Ezenwa, Gerardo, Inouye, Medina, & Xavier, 2012), but further studies and monitoring would need to be undertaken. Finally, no significant results were found in gut microbiota variation due to botfly infection of *Cuterebra* sp. Even if the high prevalence of this infection in *Peromyscus* species has been reported, these parasites have been linked to little effect on host population densities or fitness in general (Slansky, 2007). Our results confirm this trend from the microbiota perspective.

This study simulated how captive breeding programs can impact the relocation process of animals under ex situ conservation actions. We demonstrated that captive diet has an impact on the microbiota of a generalist host, even after relocation to a natural habitat. As the gut microbiota takes part in many aspects of an animal's biology, survival, and reproductive success, one should consider the microbiota aspect as well as the host's nutrition for the development of diets in captive settings. Researchers should continue to study the effect of captivity on the reintroduction process of endangered species at different scale levels: ecosystem, population, individual, and microbiota, and integrate them into management practices.

## CONFLICT OF INTEREST

The authors declare that there is no conflict of interest.

## AUTHOR CONTRIBUTIONS

**Pauline van Leeuwen:** Conceptualization (equal); data curation (equal); formal analysis (equal); investigation (equal); methodology (equal); project administration (equal); writing – original draft (lead); writing – review and editing (lead). **Gabriela F. Mastromonaco:** Funding acquisition (equal); methodology (equal); project administration (equal); resources (equal); supervision (equal); validation (equal); writing – review and editing (supporting). **Nadia Mykytczuk:** Conceptualization (supporting); data curation (supporting); funding acquisition (lead); methodology (supporting); resources (equal); supervision (supporting); validation (supporting); writing – review and editing (supporting). **Albrecht I. Schulte‐Hostedde:** Conceptualization (lead); funding acquisition (lead); methodology (supporting); project administration (equal); resources (equal); supervision (equal); validation (equal); visualization (equal); writing – original draft (equal); writing – review and editing (equal).

## Supporting information

Figure S1

Figure S1_caption

## Data Availability

Supporting information has been made available online. Final DNA sequences, ASV table, taxonomy table, and mapping file have been uploaded: Dryad https://doi.org/10.5061/dryad.wm37pvmh9.
