# Supplementary figures and images for: Effects of captivity, diet, and relocation on the gut bacterial communities of white‐footed mice
Source: Ecol Evol. 2020 Apr 3;10(11):4677–90. doi: 10.1002/ece3.6221 (PMC7297780; doi:10.1002/ece3.6221)

Generalized Unifrac distance

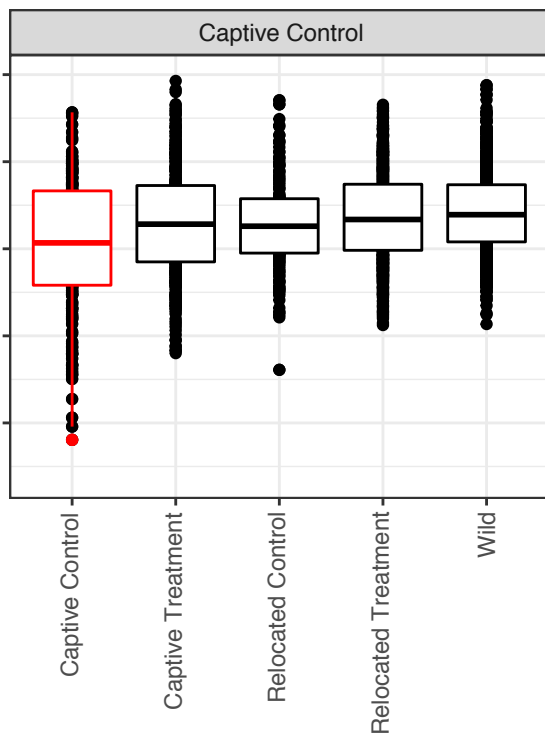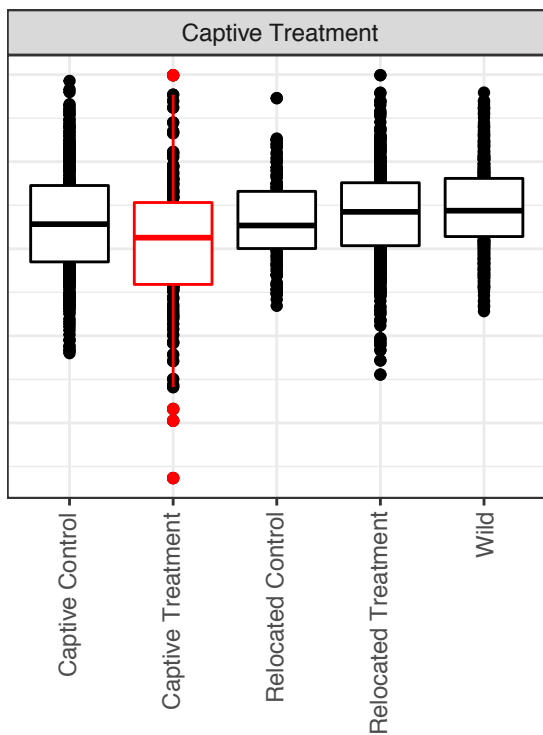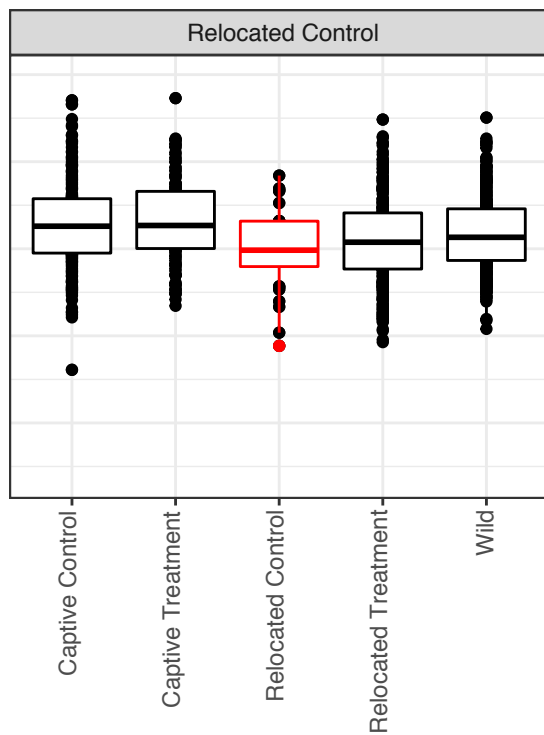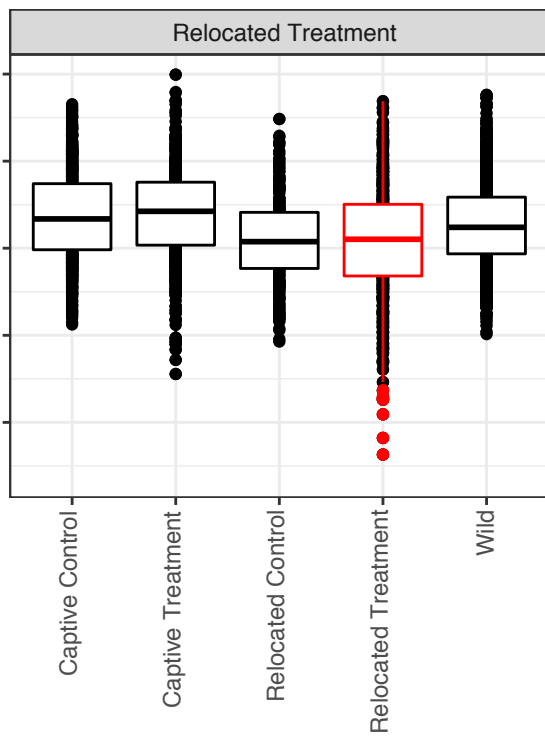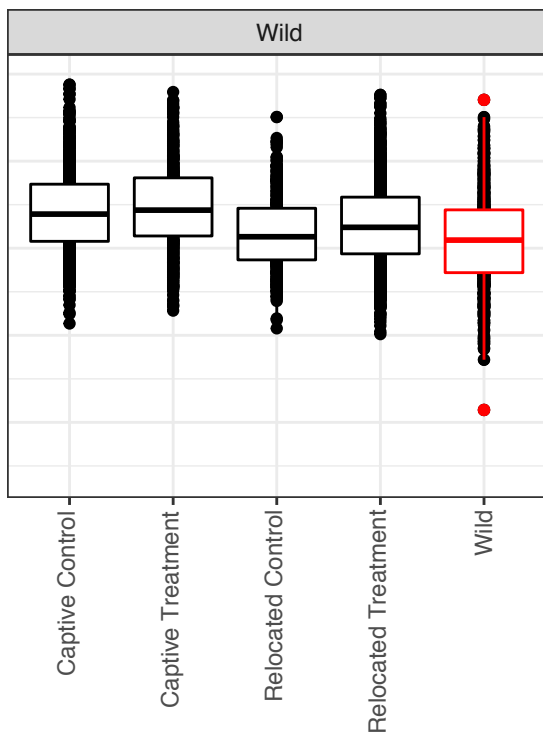

Supplement: Supplementary file 1 — Figure S1 [file ECE3-10-4677-s001.pdf]
