# Supplementary material for: Effects of captivity, diet, and relocation on the gut bacterial communities of white‐footed mice
Source: Ecol Evol. 2020 Apr 3;10(11):4677–90. doi: 10.1002/ece3.6221 (PMC7297780; doi:10.1002/ece3.6221)
Supplement: Supplementary file 2 — Figure S1_caption [file ECE3-10-4677-s002.docx]

FIGURE S1 Generalized UniFrac distance comparison boxplots of all samples between and within study groups. The boxplot is red represent the beta dispersion within the study group and in black of the said study groups between all the other groups.
